# Parsimonious root systems and better root distribution can improve biomass production and yield of soybean

**DOI:** 10.1371/journal.pone.0270109

**Published:** 2022-06-23

**Authors:** Enoch Noh, Benjamin Fallen, Jose Payero, Sruthi Narayanan

**Affiliations:** 1 Department of Plant and Environmental Sciences, Clemson University, Clemson, South Carolina, United States of America; 2 Soybean and Nitrogen Fixation Unit, USDA-ARS, Raleigh, North Carolina, United States of America; 3 Edisto Research and Education Center, Department of Agricultural Sciences, Clemson University, Blackville, South Carolina, United States of America; KGUT: Graduate University of Advanced Technology, ISLAMIC REPUBLIC OF IRAN

## Abstract

Enhancing the acquisition of belowground resources has been identified as an opportunity for improving soybean productivity worldwide. Root system architecture is gaining interest as a selection criterion in breeding programs for enhancing soil resource acquisition and developing climate-resilient varieties. Here we are presenting two novel characteristics of soybean root system architecture that improve aboveground growth and yield. Eleven selected soybean genotypes were tested under rain-fed conditions in 2019 and 2020 at two locations in South Carolina, in which one of the locations was characterized by compacted soils. The elite SC breeding line SC07-1518RR, exotic pedigree line N09-12854, and slow wilting line N09-13890 were superior genotypes in terms of biomass production, seed yield, and/or water use efficiency. Genotypes N09-12854 and N09-13890 demonstrated reduced root development (based on total root count and length), likely to restrict belowground growth and allocate more resources for shoot growth. This characteristic, which can be referred as a parsimonious root phenotype, might be advantageous for soybean improvement in high-input production systems (characterized by adequate fertilizer application and soil fertility) that exist in many parts of the world. Genotype SC07-1518RR exhibited a similar strategy: while it maintained its root system at an intermediate size through reduced levels of total root count and length, it selectively distributed more roots at deeper depths (53–70 cm). The increased root distribution of SC07-1518RR at deeper depths in compacted soil indicates its root penetrability and suitability for clayey soils with high penetration resistance. The beneficial root phenotypes identified in this study (parsimonious root development and selective root distribution in deeper depths) and the genotypes that possessed those phenotypes (SC07-1518RR, N09-12854, and N09-13890) will be useful for breeding programs in developing varieties for optimal, drought, and compacted-soil conditions.

## Introduction

Soybean [*Glycine max* (L.) Merr.] is the most important oilseed and one of the most important and affordable protein sources worldwide [1]. Soybean is the second most-planted field crop, and the second most revenue-generating crop in the United States [1]. Brazil, United States, and Argentina are the major producers of soybean in the world and together, they account for >80% of global soybean production [1]. Worldwide, soybean production is threatened by several environmental stresses, drought being the major one among them [2–8].

To date, soybean genetic improvement has primarily focused on increasing yield. However, in recent years, root architecture is gaining interest as a selection criterion in breeding programs [9–14]. Recent research on plant roots supports the reliability of incorporating root traits in crop improvement programs [15]. Lynch proposed that breeding for individual root phenotypes related to yield under stress has advantages over direct selection for yield because the underlying individual root phenes [elemental unit of the phenotype [16]] are (a) controlled by simpler genetics than that of yield, (b) are more robust and stable than yield per se, and (c) demonstrate less genotype-by-environment interaction [17]. Furthermore, recent advances in root research have identified the usefulness of an ideotype breeding strategy where beneficial root phenes from diverse sources are combined into a single elite genotype. This strategy has greater potential to enhance yields than traditional yield-based selection [15, 18, 19].

Root system architecture describes the spatial configuration of root system that depends upon root morphology, topology, and distribution [20]. Root system architecture influences crop performance under multiple environmental conditions, and present opportunities for crop improvement [17]. For example, distribution of more root length at shallow depth helps for greater tolerance to phosphorus deficiency [21], whereas distribution of more root length at deeper depth helps for drought tolerance [22, 23]. Soybean typically maintains a root system architecture in which basal, hypocotyl, and primary root production are balanced, which helps adapt to moderate drought or low-fertility conditions [24]. To further improve its drought tolerance in high-input/fertility production systems, a trait-based selection strategy to increase allocation to the primary root system would be beneficial [24]. This is primarily because the maximum rooting depth of soybean is determined by the depth of the primary root tip and the composite root length density of secondary and tertiary roots originating from the primary root [25]. From a crop improvement perspective, phenes that influence rooting depth and are under distinct genetic control would be better selection criteria in breeding programs rather than rooting depth per se [15, 16]. Such root phenes will be strongly associated with aboveground growth and yield if they reduce the metabolic costs of soil exploration [15].

Mechanical impedance in compacted soils often leads to reduced total root length and/or redistribution of root length at various depths, and thus, affects the acquisition of water and nutrients by plants [26, 27]. In the southeastern United States and many other soybean growing regions, the soybean crop is often grown on compacted soils. Furthermore, periodic droughts are common in most if not all soybean production regions, and dry soils are generally more compact [28–32]. Roots may confine to surface soil strata when they are unable to penetrate compacted soil zones [33, 34]. Genotypes may adjust their root distribution with depth in response to soil compaction [33]. However few studies have investigated distribution of roots under compaction and variability among genotypes for this trait.

Genetic mapping studies have identified quantitative trait loci (QTL) linked to root phenotypes in field crops [35–37]. These QTL’s offer opportunities for including beneficial root phenes in breeding programs through marker-assisted selection, rather than more labor-intensive root phenotyping [38, 39]. Even with the remarkable capabilities and ever decreasing cost of genotyping, breeding efforts in soybean are still constrained by the inadequate understanding of the fitness value of specific root phenotypes across diverse conditions [17, 40].

To identify root ideotypes which could be breeding targets, we need to understand how root phenotypes influence soil resource capture and plant performance in various environments [41]. Current science proposes the need for a ‘whole plant in whole soil’ approach for breeding climate-resilient crops [41]. This approach focuses on field‐scale responses of roots to various soil conditions. Unfortunately, little information is available in soybean regarding the phenotypic plasticity of roots in relation to the cultivation environment. Earlier perception of an optimum root architecture comprised of deep roots complemented with shallow lateral roots to efficiently forage for soil immobile nutrients [9, 42, 43]. It is elusive whether this dichotomy describes the beneficial root architecture under various soil conditions and whether there would be additional traits that would constitute an ideal root system [17, 40, 41, 44].

The objective of this study was to investigate whether root system characteristics are related with aboveground growth and yield of 11 selected soybean genotypes under rainfed conditions. We hypothesized that a root system architecture that optimizes root production and root distribution will improve biomass production and yield of soybean. Soybean genotypes were tested at two locations, in which one of the locations was characterized by compacted soils. The aboveground growth and performance of the genotypes were tested based on biomass production, leaf area index, seed yield, soil water depletion, and water use efficiency. Root production and root system size of the genotypes were characterized based on root count and root length.

## Materials and methods

### Plant material

The soybean genotypes tested in this study included five cultivars (Boggs, NC-Roy, NC-Raleigh, Crockett, and USDA-N8002), a germplasm line (R01-581F), and five breeding lines (N06-7023, N09-12854, N09-13890, SC-14-1127, and SC07-1518RR). More details about the genotypes are given in Table 1. A breeding line is an un-released genotype included in breeding programs, which can be released as a germplasm line or a variety [45]. A breeding line is released as a germplasm line if it has promising traits, but does not have good agronomic performance, which is necessary to be released as a variety. The soybean genotypes belonged to maturity groups (MG) V, VI, VII, and VIII (n = 1, 3, 4, and 3, respectively), which are recommended soybean maturity groups to be grown south of latitude 28°N [46, 47]. The genotypes were selected based on their unique features; for example, slow wilting (leaf wilting is delayed by several days, under drying soil conditions) and sustained nitrogen fixation under drought conditions—two major traits associated with drought tolerance of soybean [48–52]. Two genotypes have an exotic pedigree (N09-12854 and SC-14-1127). Exotic germplasm has been found to be useful for increasing genetic diversity of soybean and developing varieties with high yield and drought tolerance in the United States [49, 53, 54]. The study also included a high-yielding conventional cultivar, NC-Raleigh and an elite South Carolina breeding line, SC07-1518RR, which were developed for the southeastern production region of the United States and have produced high yields in multiple regional variety tests [55–57]. The seeds of all genotypes were obtained from Dr. Benjamin Fallen at the USDA-ARS, Raleigh, NC, USA (co-author on this paper); no permissions were necessary to collect seed samples.

**Table 1:** Characteristics of the soybean genotypes used in the study.

Genotype	Pedigree	Maturity group	Characteristics/Comments	Source of information	Geographical origin
R01-581F	Jackson x KS 4895	V	Sustained nitrogen fixation under drought	[48]	AR, United States
Boggs	G81-152 x Coker 6738	VI	Intermediate in wilting	[58]	GA, United States
N06-7023	N98-7265 x N98-7288	VI	Slow wilting	[59]	NC, United States
NC-Roy	Holiday x Brim	VI	Fast wilting	[60]	NC, United States
N09-12854	N7103 x PI408337-BB	VII	Exotic pedigree	[61]	NC, United States
N09-13890	TCPR-83 x 11136	VII	Slow wilting	[62]	NC, United States
NC-Raleigh	N85-492 x N88-480	VII	High-yielding conventional cultivar in the southeastern United States	[55]	NC, United States
SC-14-1127	NC Raleigh x PI 378696B	VII	Exotic pedigree	[59]	SC, United States
Crockett	PI 171451 x Hampton 266	VIII	Forage	[63]	TX, United States
SC07-1518RR	SC01-809RR x G99-3211	VIII	Elite South Carolina breeding line	[59]	SC, United States
USDA-N8002	N7002 x N98-7265	VIII	Slow wilting	[64]	NC, United States

### Experimental sites and plant husbandry

Field experiments were conducted in the summer (June through November) of 2019 (season-1) and 2020 (season-2) at two locations: the Piedmont Research and Education Center of Clemson University in Pendleton, SC, USA (34°37’15”N, 82°43’58”W and 255 m above sea level (a.s.l.) in 2019; 34°37’21”N, 82°44’13”W and 249 m a.s.l. in 2020) and the Pee Dee Research and Education Center of Clemson University in Florence, SC, USA (34°18’24”N, 79°44’38”W and 40 m a.s.l. in 2019; 34°18’09”N, 79°44’55”W and 40 m a.s.l. in 2020). Both Pendleton (located in the northern part of SC) and Florence (located in the south-eastern part of SC) represent major soybean producing areas in the state. Details of the experimental sites and field operations are given in supplementary material (S1 Table). The clayey soil at Pendleton was characterized by high levels of compaction [penetration resistance of 2.07 Mpa, which impose severe impedance to root growth [65, 66] at 8 cm depth], whereas the sandy soil at Florence had lower levels of compaction (penetration resistance of 2.07 Mpa at 31 cm depth). At both locations, plot size was 6.1 m by 3.0 m, and there were four rows in each plot. Field plots were arranged in a randomized complete block with five replications at Pendleton and four replications at Florence in both years. The experimental fields were bordered by four bulk rows at both locations in both years. Bulk rows were planted with the cultivar Paul at Pendleton in both years and at Florence in 2020 and with Dillon at Florence in 2019. No irrigation was applied at both locations in both years.

### Aboveground measurements: Biomass production, leaf area index, soil water depletion by crop, water use efficiency, and seed yield

In 2019, aboveground biomass was hand-harvested from five consecutive plants from the fourth row of each plot at 118, 130, and 146 days after planting (DAP) at Pendleton. Biomass was not measured in 2019 at Florence. In 2020, biomass was hand-harvested from 1 m of the fourth row of each plot at both locations. Biomass was harvested at 49, 79, 104, and 127 DAP at Pendleton and 47, 86, and 119 DAP at Florence in 2020. The central rows were not used for harvesting biomass as they were being used for collecting data on root traits and soil water content throughout the season. Each harvested row was bordered at least by four other rows or bulk rows, to avoid edge effects at both locations. Furthermore, when harvesting biomass, at least 0.25 m was avoided from the ends of the row and spaces left by previous harvests. Biomass samples were dried to constant weight at 70°C [67] to determine dry weight. Biomass was calculated on a land-area basis (kg ha^-1^), using row spacing and row length of harvested plants. For example, to express 2019- biomass dry weights at Pendleton on a land-area basis (weight per unit area) for each plot, biomass dry weight of the five consecutive plants harvested from that plot was divided by the area occupied by them (area occupied by the five consecutive plants was calculated by multiplying the row length occupied by them by row spacing). Similarly, to express 2020- biomass dry weights at both locations on a land-area basis for each plot, biomass dry weight from 1-m of harvested row length in that plot was divided by the harvested area (harvested area was calculated by multiplying the harvested row length by row spacing).

Leaf area index (LAI) was measured using a plant canopy analyzer (LAI-2200C; LI-COR Biosciences, Lincoln, NE, USA) at 134 DAP in 2019 and 78 DAP in 2020 at Pendleton and at 101 DAP in 2019 and 78 DAP in 2020 at Florence. This instrument estimates LAI as a function of canopy transmittance of diffused solar radiation [68]. Measurements in each plot consisted of a single reading of diffused radiation above the canopy and seven readings of diffused radiation below the canopy. All readings were taken within 4 min to minimize atmospheric variation. The LAI was solved analytically using the LI-COR software to obtain a single LAI value for each plot.

To measure crop water use, soil water content was measured by a neutron thermalization method using a neutron moisture meter (CPN 503 Elite^™^ HydroProbe at Pendleton and CPN 503DR HydroProbe^®^ at Florence. Both probes are manufactured by Instrotek Inc., Raleigh, NC, USA). For the neutron moisture meter to get access to the soil profile, a hollow aluminum tube with an outside diameter of 5.08 cm and length of 1.2 m with a capped bottom was inserted into the ground vertically using a tractor mounted AMS 9110 Ag Probe (AMS, Inc., American Falls, ID, USA). The aluminum access tubes were installed in the second row of each plot avoiding at least 1 m from the ends of the row. The access tubes were closed at the top by aluminum softdrink-cans to prevent water from getting into the tube. The neutron moisture meter consists of two main components, a probe that contains a source of fast neutrons and a gauge that monitors the flux of slow neutrons scattered by the soil. To measure volumetric water content, the neutron moisture meter was lowered into the access tube, where it emits fast neutrons. The fast neutrons interact with hydrogen in the soil water and thermalize (slow down) to slow neutrons. The thermal or slow neutrons are then detected by the gauge. An increase in water content results in a proportional increase in thermal neutrons that are counted by the gauge. The neutron moisture meter was calibrated locally for the specific soil types at both locations following the manufacturer’s protocol. On each soil moisture measurement date, a single count (reading) was taken by the gauge at each desired depth with 15 sec as the count time for the length of a reading. The moisture counts were divided by the average of three standard counts collected on that measurement date to obtain count ratios (count divided by standard count). Standard counts were taken with the neutron moisture meter locked in the polypropylene shielding positioned on top of the transport case. When taking standard counts, the moisture meter was at least 0.6 m away from any material that could influence the count. The count ratios were converted to volumetric water content based on a probe specific calibration equation provided by the manufacturer.

The volumetric water content (m^3^ m^–3^) was determined at 0.1, 0.2, 0.3, 0.4, 0.5, and 0.6 m depths at 118, 130, and 146 DAP at Pendleton in 2019. Data on volumetric water content were not collected at Florence in 2019. In 2020, the volumetric water content (m^3^ m^–3^) was determined at 0.15, 0.30, 0.45, 0.60, 0.75, and 0.85 m depths at both locations. The volumetric water contents were measured at 48, 79, 106, and 128 DAP at Pendleton and 46, 80, and 120 DAP at Florence in 2020. The total stored soil water (m) to specific depths (0.60 m in 2019 and 0.85 m in 2020) in each plot was estimated using individual volumetric soil water content values at various depths and the respective depth intervals with the following formula [69, 70]:

Totalstoredsoilwater(m)toadepthof0.6min2019=0.1xsumofindividualvolumetricsoilwatercontentsat0.1,0.2,0.3,0.4,0.5,and0.6mdepths (1)

Totalstoredsoilwater(m)toadepthof0.85min2020=(0.15xsumofindividualvolumetricsoilwatercontentsat0.15,0.30,0.45,0.60,and0.75mdepths)+(0.1xvolumetricsoilwatercontentat0.85mdepth) (2)

Soil water depletion (evapotranspiration) in each plot between two specific water-monitoring dates was calculated as the difference between the total stored soil water (within 0.60 m in 2019 and 0.85 m in 2020) at the two monitoring dates + precipitation during that time interval. Precipitation data pertaining to the Pendleton experimental site were obtained from the National Centers for Environmental Information (NCEI) of National Oceanic and Atmospheric Administration (NOAA), using the weather station closest to the field (Sandy Springs 2 NE, SC, US). Precipitation data pertaining to the Florence experimental site were obtained from the Clemson University’s Pee Dee Research and Education Center Weather Station at Florence (https://www.clemson.edu/cafls/research/peedee/weather.html). The water use efficiency values of each genotype for a period between 118 and 146 DAP at Pendleton in 2019, 48 and 128 DAP at Pendleton in 2020, and 46 and 120 DAP at Florence in 2020 were calculated as the ratio between biomass production and soil water depletion during the respective time interval [70]. Water use efficiency was not estimated at Florence in 2019 as data on volumetric water content were not collected at Florence in that year.

At full maturity (growth stage R8; [71]), plants were harvested for measuring seed yield. In 2019, all plants from a 1-m length of the 2^nd^ row of each plot were harvested at Pendleton on November 20^th^ (146 DAP). Harvested plants were dried to constant weight and seeds were separated and weighed. All plants from the 2^nd^ and 3^rd^ row of each plot were harvested using an Almaco SPC 20 combine (Almaco, Nevada, IA, USA) on November 18^th^ (169 DAP) at Pendleton in 2020. Similarly, all plants from the 1^st^ and 2^nd^ row of each plot were harvested using a Quantum plot combine (Wintersteiger Inc., Salt Lake City, UT, USA) on November 14^th^ (156 DAP) in 2019 and November 20^th^ (158 DAP) in 2020 at Florence. The combines directly provided the seed weights. At both locations, seed yield (kg ha^-1^) was calculated for each plot by dividing the weight of the seeds by the harvested area (harvested area was calculated using the harvested length and row spacing).

### Root imaging and measurements

Root growth dynamics were measured using a mini-rhizotron system (CI-602 Narrow Gauge Root Imager (CID-Bioscience, Camas, WA, USA) [72]. This system consists of a cylindrical scanner and acrylic clear tubes allowing for non-destructive and repeated monitoring of root growth through the crop growth season [73, 74]. For the root scanner to get access to the soil profile, a clear acrylic minirhizotron tube with 0.06 m outside diameter and 1.05 m length (CID BioScience, Camas, WA, USA) was inserted at the second row of each plot 1-m away from the row-end which is farthest from the access tube for the neutron moisture meter. The acrylic tubes were inserted at 33 DAP in 2019 and 27 DAP in 2020 at Pendleton and at 41 DAP in 2019 and 1 DAP in 2020 at Florence. To install the acrylic tubes in the soil, soil cores (diameter, 0.07 m) were taken out using a tractor-mounted AMS 9110 Ag Probe (AMS, Inc., American Falls, ID, USA) and then, into the resulting holes, the acrylic tubes were inserted (S1 Fig). Any gaps in the holes were filled with soil. The tubes were capped at the bottom. The tubes were inserted at a 45° angle from vertical, with the angle aligning parallel to the row beneath the plants. The top of the tubes was covered with a cap to prevent entry of rainwater. Images of the root system were acquired by inserting the CI-602 Narrow Gauge Root Imager into the acrylic tubes. Each image represented a 360-degree view of the root zone facing the tube and was 21.6 × 18.2 cm in size. Four images were taken per rhizotron access tube at four different depths (∼0–18, 19–35, 36–52, and 53–70 cm). Root images were analyzed using the RootSnap! Software Version 1.3.2.25 (CID BioScience, Camas, WA, USA) to get root count (total number of roots visible in the imaging area) and root length (sum of the lengths of all roots visible in the imaging area) at four different depths. Total root count and total root length were calculated per rhizotron access tube as the sum of the root count or root length values from the four images per tube. The root images were collected at 105, 120, 131, and 145 DAP in 2019 and 44, 77, 105, and 129 DAP in 2020 at Pendleton and at 129 and 154 DAP in 2019 and 44 and 80 DAP in 2020 at Florence.

### Statistical analysis

The genotypic differences in all measured aboveground traits and root traits were analyzed separately for years and locations (Neither year nor location was part of the statistical model). Analysis of variance (ANOVA) was performed on genotypes using the GLIMMIX procedure in SAS (version 9.4, SAS Institute, Cary, NC, USA) for all measured traits. The probability threshold level (α) was 0.05. Genotype was considered as a fixed effect, and replication as a random effect. The residuals from the ANOVA models were used to check for outliers. The residuals were graphed with box plots and observations with residuals outside the box plot fences were identified as possible outliers. Outliers were detected in the biomass, LAI, seed yield, soil water depletion, water use efficiency, and root trait data sets pertaining to both locations in 2019 and/or 2020. These observations were excluded from the data sets and new ANOVA were performed. Results of the original ANOVA (based on all the observations) and the new ANOVA (with possible-outlier observations excluded) were similar suggesting that outliers were not a significant issue in the data sets. Separation of least squares means was performed using the Fisher’s least significant difference (LSD) test (α = 0.05) using the LSMEANS option in the GLIMMIX procedure.

## Results

### Environmental conditions

**Fig 1:**
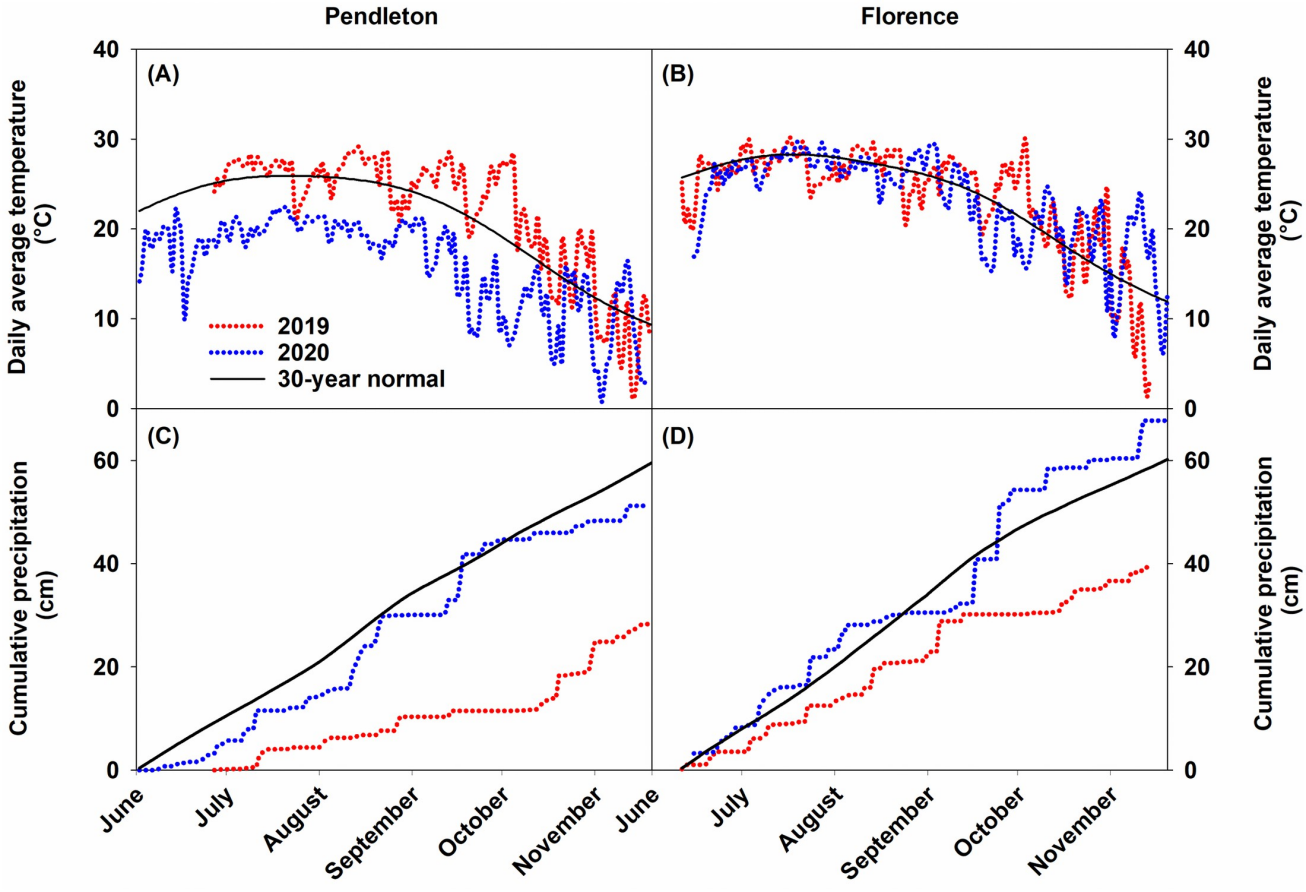
Daily average temperatures (A&B) and cumulative precipitation (C&D) during the soybean growing seasons at Pendleton, SC and Florence, SC in 2019 and 2020 in comparison with the historic weather data (daily average temperature normals for a period of 30 years from 1991 to 2020 in panels A&B and cumulative precipitation normals for the same 30-year period in panels C&D). The soybean growing seasons spanned from June 27^th^ to November 20^th^ in 2019 and June 2^nd^ to November 18^th^ in 2020 at Pendleton and June 11^th^ to November 14^th^ in 2019 and June 15^th^ to November 20^th^ in 2020 at Florence. Temperature and precipitation data pertaining to the Pendleton experimental site were obtained from the National Centers for Environmental Information (NCEI) of National Oceanic and Atmospheric Administration (NOAA), using the weather station closest to the field (Sandy Springs 2 NE, SC, US). Temperature and precipitation data pertaining to the Florence experimental site were obtained from the Clemson University’s Pee Dee Research and Education Center Weather Station at Florence.

Fig 1 shows air temperature and precipitation data during the crop growing seasons at Pendleton and Florence in 2019 and 2020, in comparison with the 30-year climate normals. Daily average temperatures were higher than that of the 30-year normal during 4–26, 41–57, 66–84, 87–108, and 121–127 DAP at Pendleton in 2019, 72–81, 86–91, 114–123, 126–137, and 143–154 DAP at Florence in 2019, and 57–66, 68–73, 87–98, 105–120, and 136–142 DAP at Florence in 2020 (Fig 1A). The crop growing seasons were significantly drier than normal based on the 30-year historic precipitation data, at Pendleton in 2019 and 2020 and at Florence in 2019 (Fig 1B). Total precipitation was 28 cm during the 146-d period at Pendleton in 2019, 51 cm during the 169-d period at Pendleton in 2020, 39 cm during the 156-d period at Florence in 2019, and 68 cm during the 158-d period at Florence in 2020 (Fig 1B). Although it depends on maturity group and location, soybean plants require ~ 75 cm of water during the growing season to achieve the maximum potential yield [75]. Hence, 2019 can be considered as a drought season for soybean crop at both locations. The distribution of precipitation was suboptimal at both locations in 2019. At Florence, a hurricane occurred on 9/4/2019 (85 DAP), which brought in 6 cm rain. After that, Florence experienced a long dry spell for a period of 27 d from 09/07/2019 (88 DAP) to 10/04/2019 (115 DAP). The only precipitation during this period at this location was a 1-cm rainfall at 94 DAP. A long dry spell occurred at Pendleton for a period of 37 d from 08/29/2019 (63 DAP) to 10/05/19 (100 DAP). The only precipitation during this period at this location was a 1-cm rainfall at 79 DAP.

### Aboveground traits: Biomass production, leaf area index, seed yield, soil water depletion by crop, and water use efficiency

**Fig 2:**
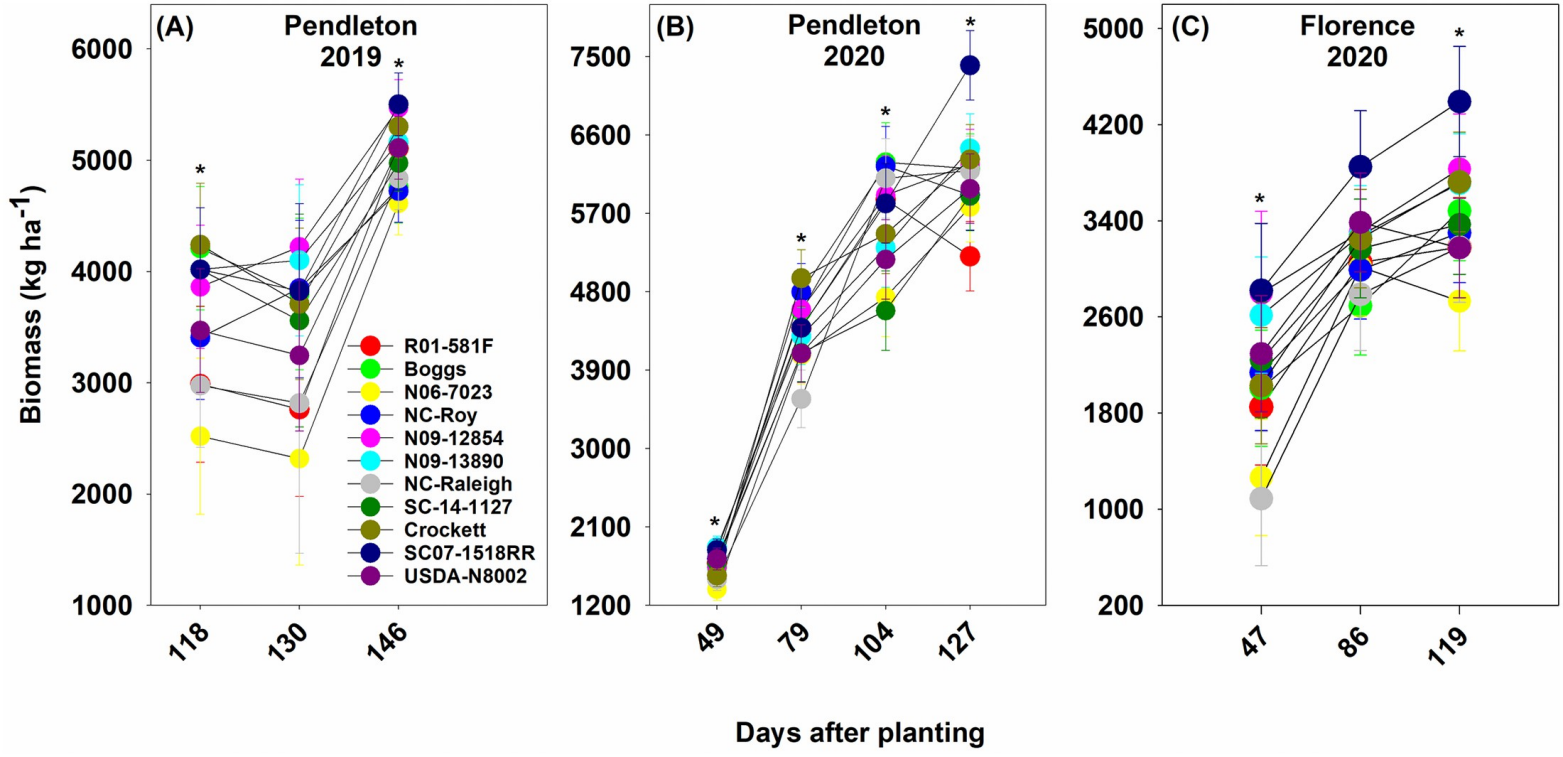
Biomass production of the soybean genotypes at Pendleton (2019, Panel A and 2020, Panel B) and Florence (2020, Panel C) in South Carolina. Biomass was not measured at Florence in 2019. Asterisk shows differences among genotypes based on the Fisher’s least significant difference (LSD) test at α = 0.05. Most genotypes reached R4 growth stage by 118 days after planting (DAP) and R7 or R8 growth stage by 146 DAP at Pendleton in 2019. Most genotypes reached V8, R4, R5, and R6 growth stages by 49, 79, 104, 127 DAP, respectively, at Pendleton in 2020. Most genotypes reached V7, R5, and R7 growth stages by 47, 86, and 119 DAP, respectively, at Florence in 2020. Growth stages were determined following Fehr and Caviness [71].

**Fig 3:**
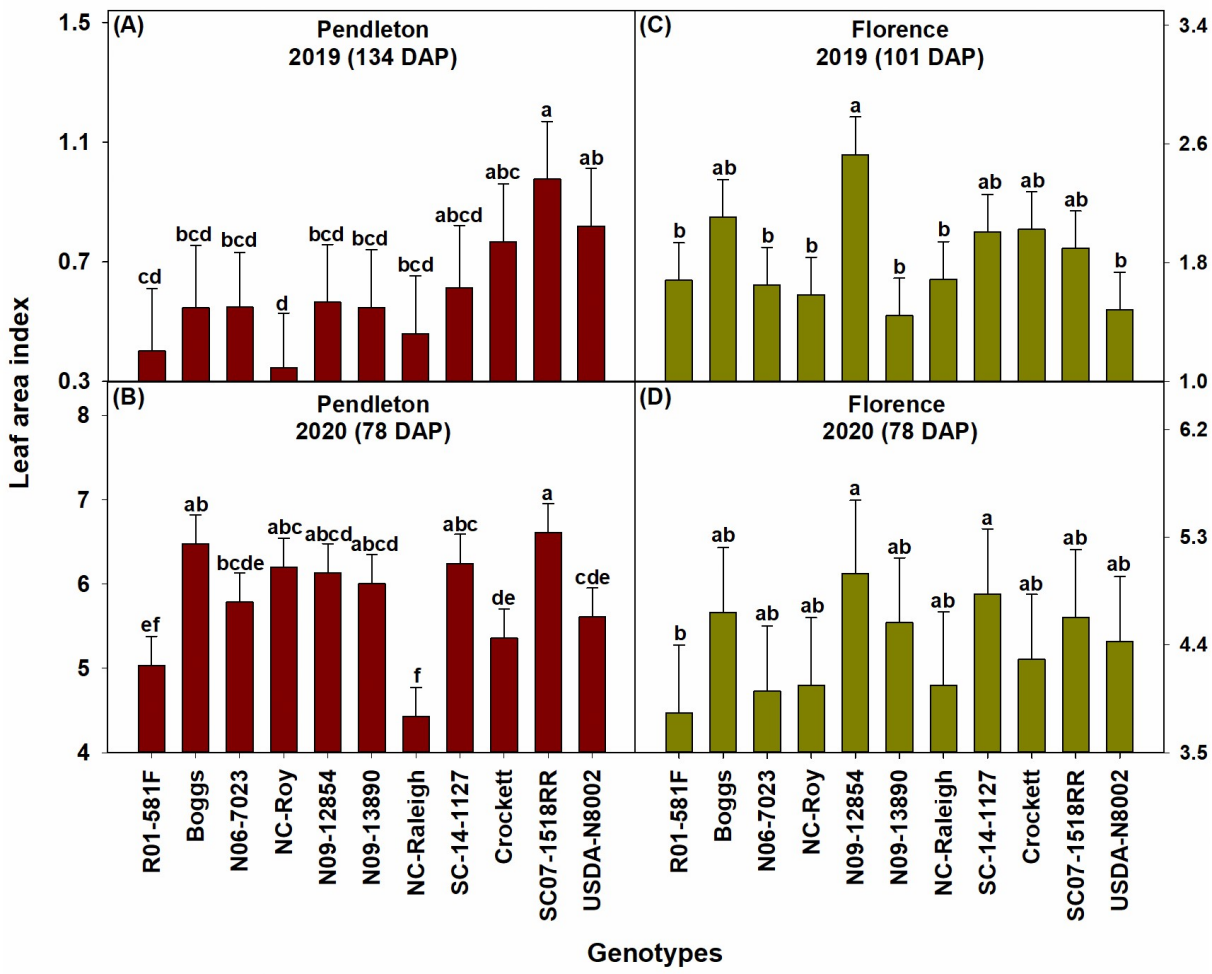
Leaf area index of the soybean genotypes at Pendleton and Florence in 2019 and 2020. Bars represent least squares means and error bars represent standard errors. Least squares means with different letters are significantly different according to the Fisher’s least significant difference (LSD) test at α < 0.05. DAP–days after planting. Most genotypes were at R5 or R6 growth stage by 134 DAP at Pendleton in 2019. Most genotypes reached R4 growth stage by 78 DAP at Pendleton in 2020, R2 growth stage by 101 DAP at Florence in 2019, and R4 growth stage by 78 DAP at Florence in 2020. Growth stages were determined following Fehr and Caviness [71].

**Fig 4:**
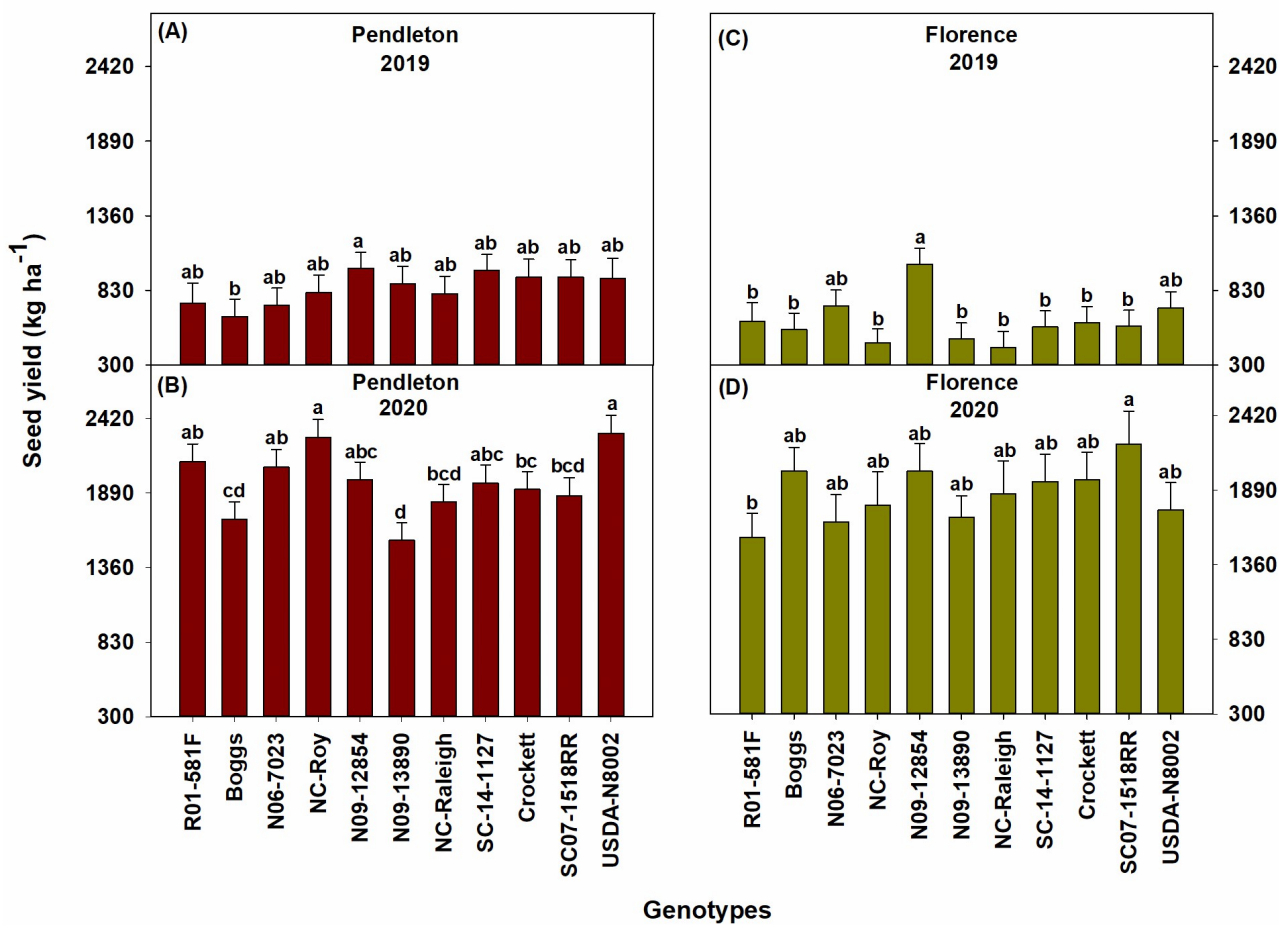
The seed yield of the soybean genotypes at Pendleton and Florence in 2019 and 2020. Seeds were harvested at full maturity [growth stage, R8; [71]] at 146 and 169 days after planting at Pendleton in 2019 and 2020, respectively, and 156 and 158 days after planting at Florence in 2019 and 2020, respectively. Bars represent least squares means and error bars represent standard errors. Least squares means with different letters are significantly different according to the Fisher’s least significant difference (LSD) test at α < 0.05.

**Fig 5:**
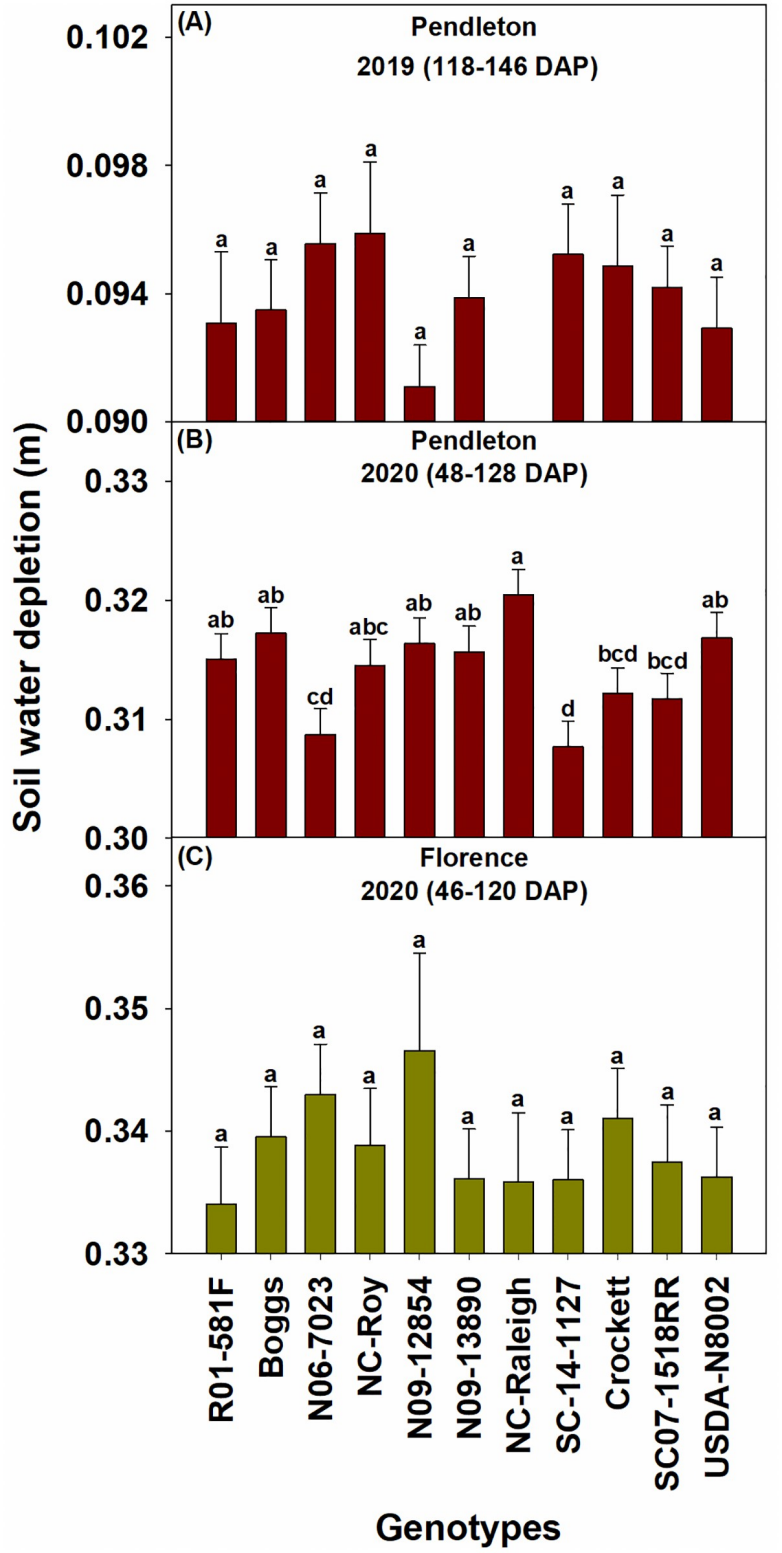
Soil water depletion by the soybean genotypes at Pendleton (2019, Panel A and 2020, Panel B) and Florence (2020, Panel C) in South Carolina. Soil water depletion was not measured at Florence in 2019. The missing data point at Pendleton in 2019 (NC-Raleigh) was due to rain damage to the access tubes installed in the plots for soil moisture measurement. Least squares means with different letters are significantly different according to the Fisher’s least significant difference (LSD) test at α < 0.05. DAP–days after planting. Most genotypes reached R5 or R6 growth stage by 130 DAP and R7 or R8 growth stage by 146 DAP at Pendleton in 2019. Most genotypes reached V8 and R6 growth stages by 48 and 128 DAP, respectively, at Pendleton in 2020. Most genotypes reached V7 and R7 growth stages by 46 and 120 DAP, respectively, at Florence in 2020. Growth stages were determined following Fehr and Caviness [71].

**Fig 6:**
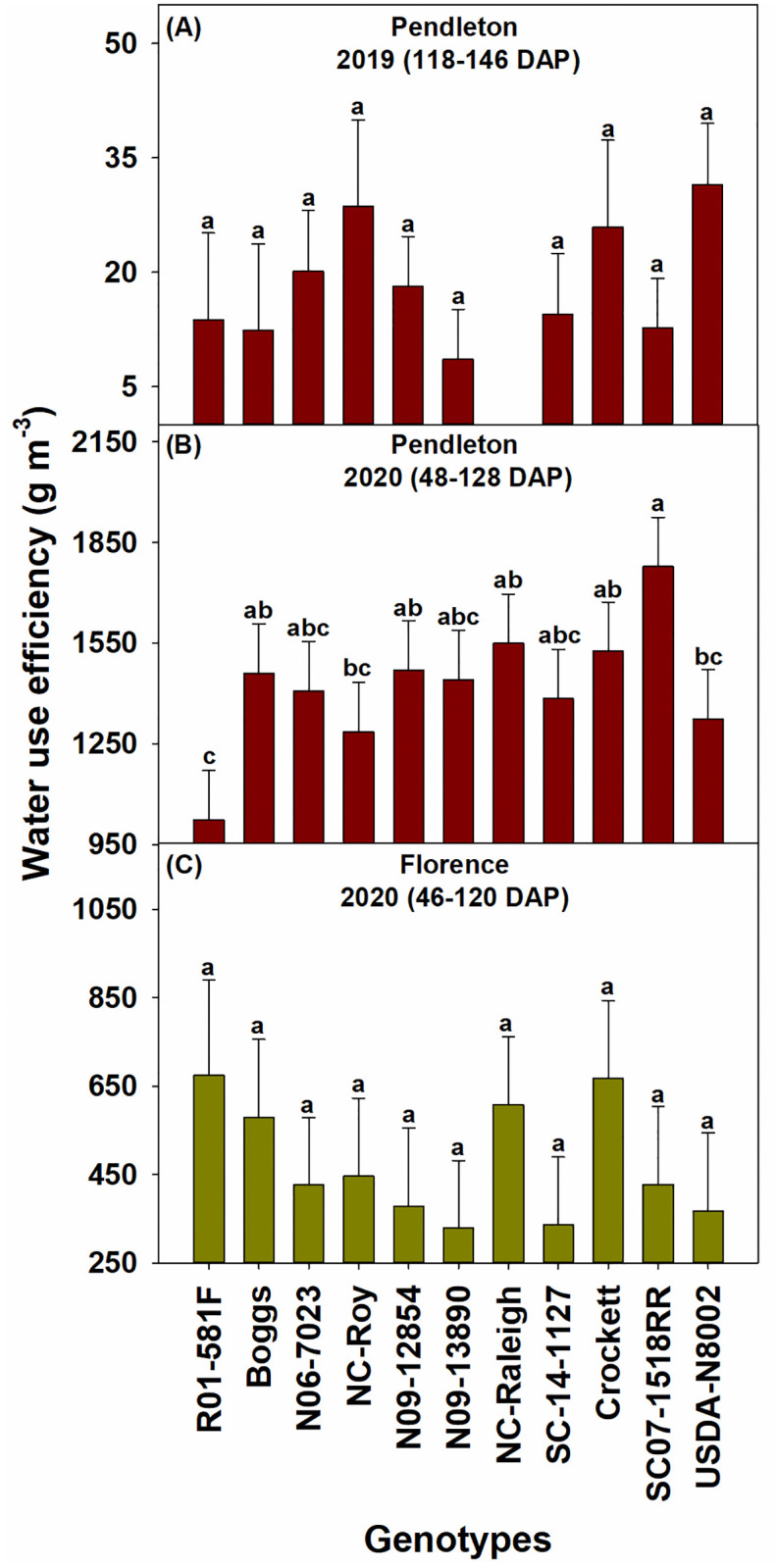
Water use efficiency of the soybean genotypes at Pendleton (A) in 2019 and 2020 (B) and Florence in 2020 (C). Water use efficiency was not measured at Florence in 2019. Least squares means with different letters are significantly different according to the Fisher’s least significant difference (LSD) test at α < 0.05. DAP–days after planting. Most genotypes reached V8 and R6 growth stages by 48 and 128 DAP, respectively, at Pendleton in 2020. Most genotypes reached V7 and R7 growth stages by 46 and 120 DAP, respectively, at Florence in 2020. Growth stages were determined following Fehr and Caviness [71].

The drought conditions in 2019 affected the performance of all genotypes in that year, which was reflected in the lower values of biomass, leaf area index, and seed yield of genotypes in 2019 than in 2020. Overall, the elite SC breeding line SC07-1518RR, the exotic pedigree line N09-12854, slow wilting line N09-13890, and forage variety Crockett had relatively high biomass during the seasons at both locations (Fig 2). Genotypes SC07-1518RR and N09-12854 also had high leaf area index at both locations (Fig 3). N09-12854 was also one among the genotypes with the highest seed yield at both locations (Fig 4). SC07-1518RR and Crockett had either relatively high or intermediate seed yield (Fig 4). N09-13890 was one of the genotypes with high seed yield at Pendleton in 2019 and at Florence in 2020 (Fig 4). We also measured soil water depletion by genotypes between the first and last measurement dates of biomass production. We found that the genotypes that produced high biomass and/or seed yield: SC07-1518RR, N09-12854, N09-13890, and Crockett did not deplete more soil water than the other genotypes, indicating that they just used equal amounts of water as the other genotypes (Fig 5). This result demonstrated that the superior performance of these genotypes in terms of biomass and/or seed yield was not the result of increased water use. The same genotypes (SC07-1518RR, N09-12854, N09-13890, and Crockett) also ranked among the best for water use efficiency when grown under clayey soil conditions at Pendleton in 2020 (Fig 6). Genotypes did not differ for water use efficiency when grown under sandy soil conditions at Florence or in the drought year of 2019 at Pendleton (Fig 6). Water use efficiency values of all genotypes were lower in the drought year of 2019 than in 2020 due to lower biomass in 2019.

### Root system development

**Fig 7:**
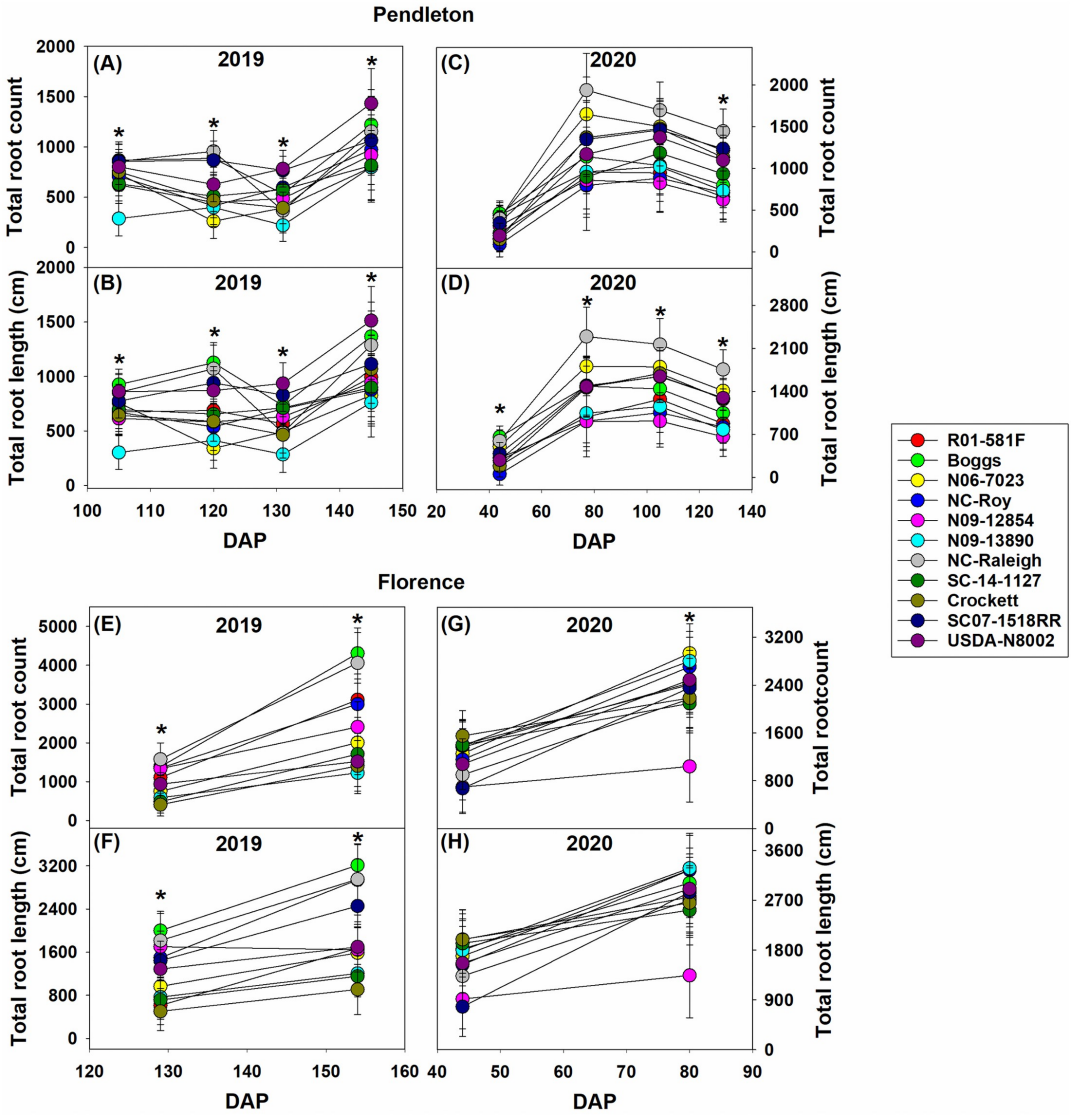
Changes in total root count and total root length of the soybean genotypes in 2019 and 2020 at Pendleton and Florence. Asterisk shows differences among genotypes based on the Fisher’s least significant difference (LSD) test at α = 0.05. DAP–days after planting. Total root count is the total number of roots visible in the imaging area of the CI-602 root imager. Total root length is the sum of the lengths of all roots visible in the imaging area of the CI-602 root imager. Most genotypes reached R1 or R2 growth stage by 105 DAP, R4 growth stage by 120 DAP, R5 or R6 growth stage by 131 DAP, and R7 or R8 growth stage by 145 DAP at Pendleton in 2019. Most genotypes reached V7, R4, R5, and R6 growth stages by 44, 77, 105, 129 DAP, respectively, at Pendleton in 2020. Most genotypes reached R6 growth stages by 129 DAP and R7 or R8 growth stage by 154 DAP at Florence in 2019. Most genotypes reached V7 and R4 growth stages by 44 and 80 DAP, respectively, at Florence in 2020. Growth stages were determined following Fehr and Caviness [71].

Root system development was measured at multiple times during crop development at both locations and in both years. Two root traits that define the amount of roots produced and size of the root system: total root count and total root length, respectively are presented in Fig 7. The root growth was greater by approximately twofold in the sandy soil at Florence, compared to that in the clayey/compacted soil at Pendleton. Differences in root traits among genotypes became more prominent toward the later part of the growth cycle (≥80 DAP) at both locations (Fig 7). Overall, Boggs, USDA-N8002, and NC-Raleigh had relatively greater and N09-13890 had relatively lower total root count and total root length than the other genotypes at Pendleton in 2019 (Fig 7A & 7B). Other genotypes were intermediate. In 2020, NC-Raleigh had relatively greater and N09-12854 and N09-13890 had relatively lower total root count and total root length than the other genotypes at Pendleton (Fig 7C & 7D), and other genotypes were intermediate. At Florence, Boggs and NC-Raleigh had relatively greater and Crockett and N09-13890 had relatively lower total root count and total root length than the other genotypes in 2019 (Fig 7E & 7F). In 2020, N09-12854 had relatively lower total root count and total root length than the other genotypes at Florence (Fig 7G & 7H). Other genotypes were more or less similar in terms of these root traits.

### Root production and root system size at various depths

**Fig 8:**
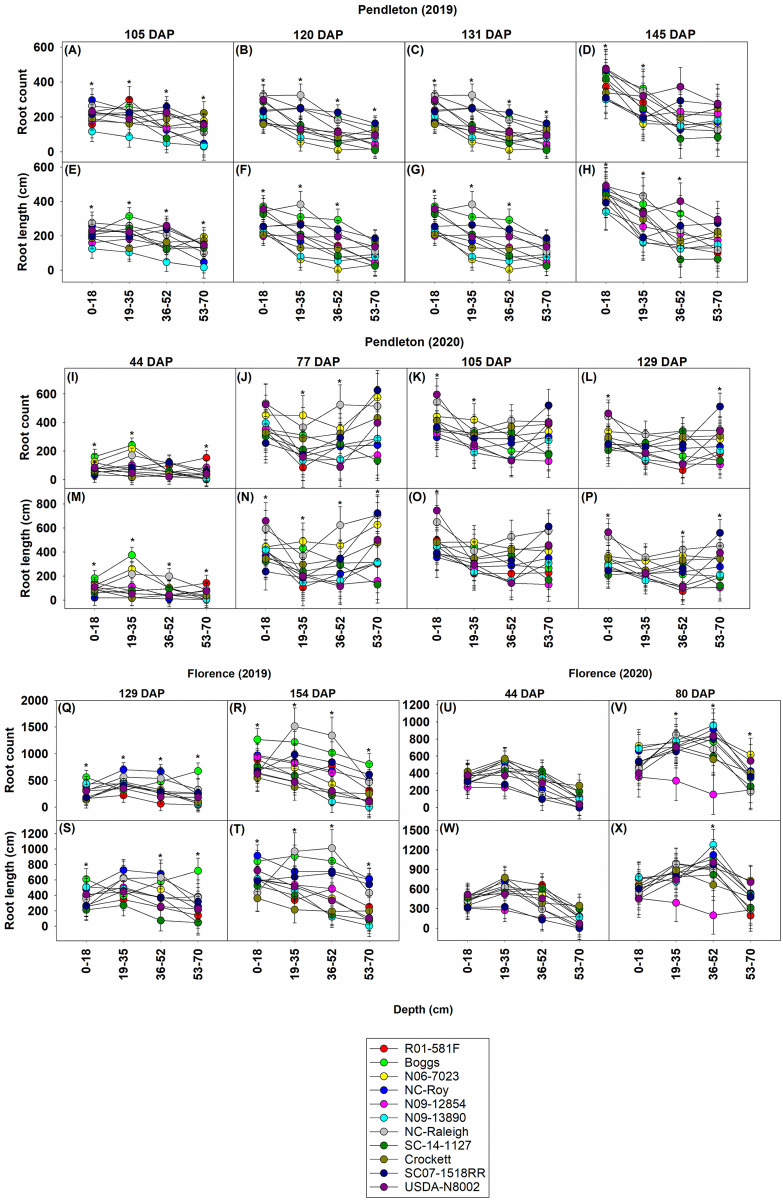
Root count and total root length of soybean genotypes at various depths at Pendleton and Florence in 2019 and 2020. Root count is the number of roots visible in the imaging area of the CI-602 root imager. Root length is the sum of the lengths of all roots visible in the imaging area of the CI-602 root imager. Asterisk shows differences among genotypes based on the Fisher’s least significant difference (LSD) test at α = 0.05. DAP–days after planting. Most genotypes reached R1 or R2 growth stage by 105 DAP, R4 growth stage by 120 DAP, R5 or R6 growth stage by 131 DAP, and R7 or R8 growth stage by 145 DAP at Pendleton in 2019. Most genotypes reached V7, R4, R5, and R6 growth stages by 44, 77, 105, 129 DAP, respectively, at Pendleton in 2020. Most genotypes reached R6 growth stage by 129 DAP and R7 or R8 growth stage by 154 DAP at Florence in 2019. Most genotypes reached V7 and R4 growth stages by 44 and 80 DAP, respectively, at Florence in 2020. Growth stages were determined following Fehr and Caviness [71].

We also estimated root count and root length at various depths: ~ 0–18, 19–35, 36–52, and 53–70 cm. SC07-1518RR showed relatively uniform distribution of roots at various depths in terms of root count and root length at Pendleton in 2019, while multiple genotypes showed decreases in these root traits at deeper depths (53–70 cm) (Fig 8A–8H). SC07-1518RR was one among the genotypes that possessed the highest values for root count and root length at 53–70 cm depth (Fig 8A–8H). Furthermore, this genotype demonstrated a significant increase in root distribution at deeper depths (≥36 cm) than at shallower depths at the end of the season (145 DAP); 52 and 39% increase in root count and 36 and 45% increase in root length at 36–52 cm and 53–70 cm depths, respectively, compared to those at 19–35 cm depth (Fig 8D & 8H). N09-12854 was low or intermediate in terms of root count and root length at all depths (Fig 8A–8H). The root count and root length of NC-Raleigh were high until the depth of 35 cm, but they decreased in deeper depths. Overall, N06-7023 and N09-13890 had low root count and root length at all depths (Fig 8A–8H). In 2020, NC-Raleigh generally had the highest root count and root length at all depths, except 53–70 cm, at Pendleton (Fig 8I–8P). SC07-1518RR was similar to other genotypes in terms of root count and root length at shallower depths (0–52 cm), but it increased these root traits at deeper depths (53–70 cm) and had the highest values for root count and root length at deeper depths (Fig 8I–8P). N09-12854 showed relatively low root count and root length at all depths (Fig 8I–8P).

At Florence, NC-Raleigh showed high root count and root length between 19 and 52 cm depth, but low root count and root length at the first 18 cm and after 52 cm depth (Fig 8Q–8T). N09-12854 was intermediate in terms of root count and root length at all depths (Fig 8Q–8T). SC07-1518RR was intermediate in terms of root count and root length at depths ≤52 cm, but was one among the genotypes with high values for these root traits at deeper depths (53–70 cm) (Fig 8Q–8T). In 2020, at the same location, SC07-1518RR was just intermediate or low in root count and root length at all depths (Fig 8U–8X). N09-12854 also showed low root count and root length at all depths (Fig 8U–8X).

## Discussion

Our results demonstrated novel characteristics related with root production and distribution that will influence biomass production and yield formation in soybean. The soybean genotypes tested in this study included cultivars and breeding/germplasm lines selected based on traits related with drought tolerance (slow wilting and sustained nitrogen fixation under drought), exotic pedigree, and elite performance in South Carolina. The elite SC breeding line SC07-1518RR, exotic pedigree line N09-12854, and slow wilting line N09-13890 were superior genotypes in terms of biomass production and/or yield at both locations and in both years (Fig 2). These genotypes exhibited some interesting root-related mechanisms that might have potentially contributed to increased biomass production and yield formation.

N09-12854 was one of the genotypes with low values for total root count and total root length at both locations in 2020, which was a relatively normal year in terms of precipitation (Fig 7). In the same year, N09-12854 was one among the genotypes with high values for leaf area index (Fig 3). This suggests that this genotype favored aboveground growth over belowground growth when precipitation and water availability were normal. Furthermore, N09-12854 was one of the genotypes with high biomass production and seed yield at both locations in 2020 (Figs 2 & 4). Increased aboveground growth, and thus increased biomass production, while restricting belowground growth helped this genotype possess high values for water use efficiency without increasing water use (Figs 5 & 6). Since soil water depletion by N09-12854 was similar to that by other genotypes (Fig 5), the increased biomass and seed yield of N09-12854 were not the results of increased water use. These observations support the parsimonious root hypothesis, which refers to reduced root development that would be advantageous for annual crops grown for seed yield in high input production systems [76]. In such production systems, application of fertilizers, herbicides, and pesticides and other crop management methods have minimized crop growth limitations from scarcity of nutrients (especially nitrogen and phosphorous), root loss due to biotic stresses, and root competition with weeds [76, 77]. Thus, rather than a prolific root system, a parsimonious root phenotype that optimize water capture by reducing investments in cells, tissues, and organs with unfavorable cost/benefit ratio would be more advantageous in high-input production systems. Parsimonious architectural phenotypes of annual crops grown for seed yield include reduced number of axial roots, reduced lateral root length and density, and loss of roots that do not contribute to water capture. Parsimonious anatomical phenotypes include reduced cortical cell file number and reduction of cortical parenchyma through formation of aerenchyma and senescence [76]. Many of these characteristics directly influence root morphological traits such as total root length. Thus, if water availability is not limiting crop growth in the high input production system, plants do not have to partition increased levels of assimilates belowground to increase root production. Instead, they can selectively partition the assimilates aboveground to increase effective photosynthetic area, which can contribute to increased seed yield.

In 2019, which was a drought year, N09-12854 had intermediate values for total root count and total root length. This suggests that when water was scarce, this genotype increased its root production and root system size to maintain resource capture. The modified root system might have helped this genotype maintain aboveground growth as it was one among the best genotypes in terms of biomass production, leaf area index, and seed yield at both locations in 2019 (Figs 2–4).

The slow wilting line N09-13890 had low values for total root count and root length. This genotype was one of the best genotypes in terms of biomass production and water use efficiency. It also demonstrated low values for soil water depletion (Fig 5), indicating low water use. Parsimonious root systems often associate with moderate water acquisition capacity, which in turn results in parsimonious water use and high water use efficiency, and can help conserve soil water until maturity [24]. Our results suggest that a parsimonious root system (reduced root production and root system size) may be related with the slow wilting trait of N09-13890.

SC07-1518RR was one of the best genotypes in terms of biomass production and leaf area index at both locations in both years (Figs 2 & 3). It also had relatively high or intermediate seed yield (Fig 4). But, it possessed only intermediate values for total root count and total root length at both locations and in both years (Fig 7). This suggests that similar to N09-12854 and N09-13890, this genotype also did not produce more roots and increase root system size to support aboveground growth. However, SC07-1518RR distributed more roots in the deeper depths (53–70 cm) (Fig 8). At Pendleton, this genotype generally had the largest numerical values for root count and root length at 53–70 cm depth throughout the season in both years. The clayey soil of Pendleton experimental field was characterized by high compaction (penetration resistance of 2.07 MPa just at 8 cm depth). Soil compaction generally reduces rooting depth in plants and modifies root distribution by enforcing greater root length densities closer to the soil surface [78]. Typically, root elongation is halved in compacted soils with penetration resistances of 0.8–2 MPa, in the absence of water stress [66]. Only the genotypes with high root penetrability of compacted soils will exhibit roots at deeper depths, and our data suggest that SC07-1518RR is one such genotype. The root penetrability of SC07-1518RR is supported by our previous research in which the same genotype penetrated a synthetic hardpan placed in the growth columns under controlled environmental conditions [79]. Deep rooting is especially advantageous for plants in drought years like 2019. Greater rooting depth has been found to improve yields when subjected to terminal drought in multiple species including soybean [80], chickpea (*Cicer arietinum* L.) [81, 82], and groundnut (*Arachis hypogaea* L.) [83, 84]. In the sandy soil of Florence, SC07-1518RR was one among the genotypes with the highest values for root count and root length in the deeper depths (53–70 cm) in the drought year, 2019 (Fig 8). However, it appears that, in the same location when precipitation was normal in 2020, this genotype did not invest much in root system, and its root count and root length values were low or intermediate at all depths (Fig 8). Our results suggest that SC07-1518RR possesses some interesting characteristics related with root system development and root distribution in the soil profile. This genotype does not increase total root production and root system size at the expense of aboveground growth (leaf area and biomass). Instead, it improves root distribution in the soil profile, and can selectively distribute roots in the deeper soil profile, especially when water availability is low in the upper soil profile. Under compacted soil conditions, when most genotypes are unable to penetrate to deep soil layers, this genotype is able to do that and preferentially distribute roots in deeper depths. This trait will be advantageous for improving soybean performance in clayey/compacted soils, particularly under drought conditions.

Vanhees et al. [78] found that maize (*Zea mays* L.) rooting depth is not dependent on the total amount of roots formed and root length under compacted soil conditions as both large and parsimonious root systems reach similar depths. This is because the ability of roots to grow through compacted soil is not dependent on the total amount of roots formed, instead some morphological and anatomical traits. It was found in maize that the frictional resistance to root growth in compacted soils is reduced by sloughing of root cap cells and mucilaginous exudates produced by roots [85–87]. Colombi et al. [88] found that smaller root tip radius-to-length ratio helps wheat roots penetrate compacted soils. Root tip traits that decrease frictional resistance and axial cell wall tension are also beneficial to root penetration [66]. Root anatomical traits also help overcome mechanical impedance. Examples are greater cortical cell diameter that reduces energy costs under impeded conditions [89] and smaller outer cortical cells that prevent buckling and facilitate penetration of harder layers [90].

NC-Raleigh exhibited a contrasting root phenotype to that of SC07-1518RR and N09-12854. NC-Raleigh had relatively higher values for total root count and total root length (Fig 7). At the same time, it produced relatively low or intermediate amounts of biomass, leaf area, and/or seed yield and had similar values for water use efficiency as other genotypes (Figs 2–4 and 6). These results support the postulate that the reduced root development of SC07-1518RR, N09-12854, and N09-13890 might have helped reduce metabolic cost for root production and enhance allocation of resources to shoot growth, which are advantages of a parsimonious root phenotype. These effects would be more apparent under high input production systems that typically exist in the United States, where genotypes with parsimonious root phenotypes will exhibit enhanced conversion of soil resources to yield [76, 91]. Such reduced root development could be advantageous for drought tolerance as well in high-input production systems [76].

## Conclusions

Our results demonstrated two novel characteristics of soybean root system architecture that can improve aboveground growth and yield. The elite SC breeding line SC07-1518RR, exotic pedigree line N09-12854, and slow wilting line N09-13890 were superior genotypes in terms of biomass production, seed yield, and/or water use efficiency. Genotypes N09-12854 and N09-13890 demonstrated reduced root development, likely to restrict belowground growth and allocate more resources for shoot growth. This characteristic, which can be referred as a parsimonious root phenotype, might be advantageous for soybean improvement in high input production systems that typically exist in the United States. Genotype SC07-1518RR exhibited a similar strategy: while it maintained its root system at an intermediate size, it selectively distributed more roots at deeper depths (53–70 cm). The increased root distribution of SC07-1518RR at deeper depths in the compacted soil at Pendleton indicates the root penetrability of this genotype and its suitability for clayey soils with high penetration resistance. The beneficial root phenotypes identified in this study (parsimonious root development and selective root distribution in deeper depths) and the genotypes that exhibited those phenotypes (SC07-1518RR, N09-12854, and N09-13890) will be useful for breeding programs in developing varieties for optimal, drought, and compacted-soil conditions. Future studies are warranted to confirm the present results with more genotypes belonging to different root architectural groups (parsimonious or normal) under multiple locations.

## Supporting information

S1 Fig Root imaging and analysis. Taking out soil cores using a tractor-mounted AMS 9110 Ag Probe (AMS, Inc., American Falls, ID, USA) to install access tubes for the CI-602 root imager (A). The CI-602 root imager inserted into the acrylic access tubes for root imaging (B). Collection of root images in a tablet computer connected to the CI-602 root imager (C). Analysis of a root image using the Rootsnap! Software Version 1.3.2.25 (CID BioScience, Camas, WA, USA) (D).
(DOCX)

S1 Table Details of field operation. (XLSX)

S2 Table Raw data. (XLSX)
