# Supplementary material for: Parsimonious root systems and better root distribution can improve biomass production and yield of soybean
Source: PLoS One. 2022 Jun 23;17(6):e0270109. doi: 10.1371/journal.pone.0270109 (PMC9223306; doi:10.1371/journal.pone.0270109)
Supplement: S1 Fig — Taking out soil cores using a tractor-mounted AMS 9110 Ag Probe (AMS, Inc., American Falls, ID, USA) to install access tubes for the CI-602 root imager (A). The CI-602 root imager inserted into the acrylic access tubes for root imaging (B). Collection of root images in a tablet computer connected to the CI-602 root imager (C). Analysis of a root image using the Rootsnap! Software Version 1.3.2.25 (CID BioScience, Camas, WA, USA) (D). (DOCX) [file pone.0270109.s001.docx]

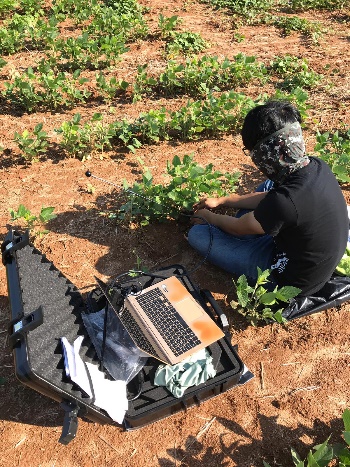

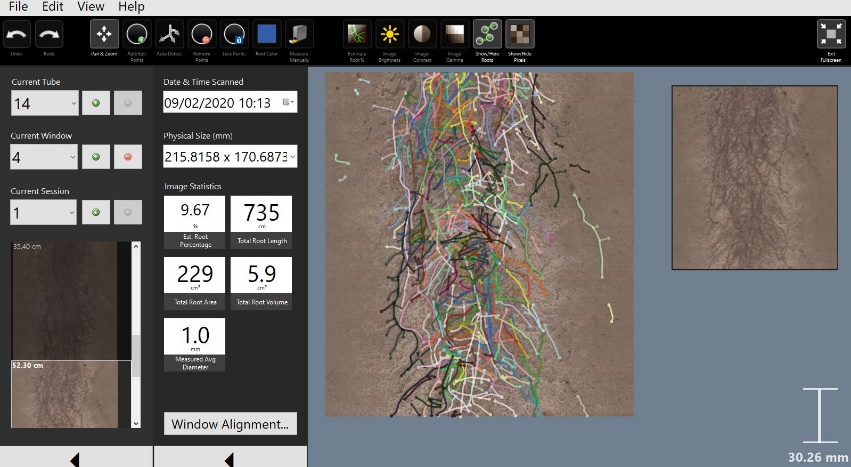

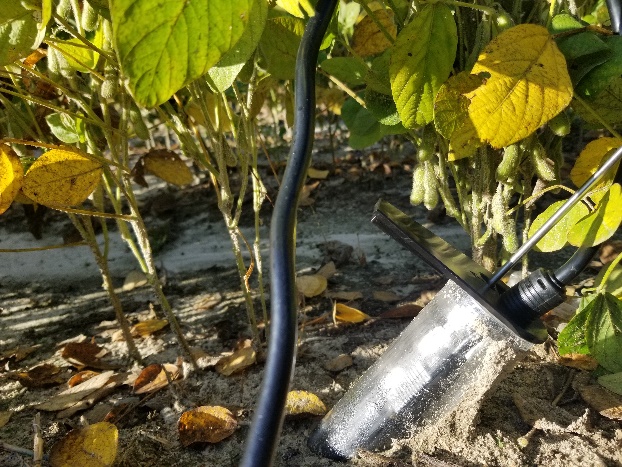

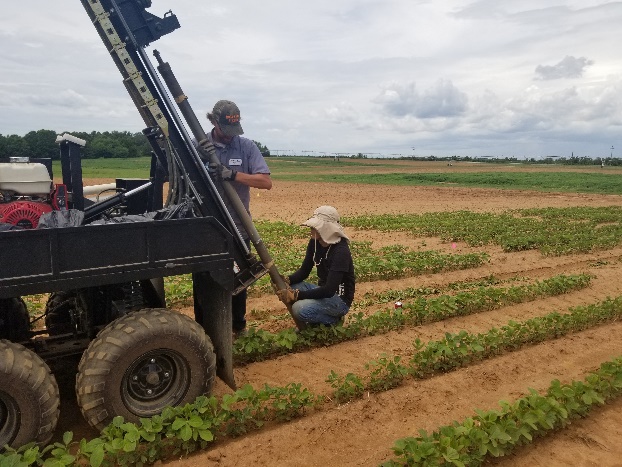


**(D)**

**(C)**

**(B)**

**(A)**

**Fig S1.** Root imaging and analysis. Taking out soil cores using a tractor-mounted AMS 9110 Ag Probe (AMS, Inc., American Falls, ID, USA) to install access tubes for the CI-602 root imager (A). The CI-602 root imager inserted into the acrylic access tubes for root imaging (B). Collection of root images in a tablet computer connected to the CI-602 root imager (C). Analysis of a root image using the Rootsnap! Software Version 1.3.2.25 (CID BioScience, Camas, WA, USA) (D).
